# Composition, Buoyancy Regulation and Fate of Ice Algal Aggregates in the Central Arctic Ocean

**DOI:** 10.1371/journal.pone.0107452

**Published:** 2014-09-10

**Authors:** Mar Fernández-Méndez, Frank Wenzhöfer, Ilka Peeken, Heidi L. Sørensen, Ronnie N. Glud, Antje Boetius

**Affiliations:** 1 HGF-MPG Group for Deep Sea Ecology and Technology, Alfred-Wegener-Institut Helmholtz-Zentrum für Polar- und Meeresforschung, Bremerhaven, Germany; 2 HGF-MPG Group for Deep Sea Ecology and Technology, Max Planck Institute for Marine Microbiology, Bremen, Germany; 3 Polar Biological Oceanography, Alfred-Wegener-Institut Helmholtz-Zentrum für Polar- und Meeresforschung, Bremerhaven, Germany; 4 MARUM, Center for Marine Environmental Sciences, University of Bremen, Bremen, Germany; 5 Nordic Centre for Earth Evolution, University of Southern Denmark, Odense, Denmark; 6 Greenland Climate Research Centre, Nuuk, Greenland; 7 Marine Biogeochemistry, Scottish Association for Marine Science, Oban, United Kingdom; 8 Arctic Research Centre, University of Aarhus, Aarhus, Denmark; Auckland University of Technology, New Zealand

## Abstract

Sea-ice diatoms are known to accumulate in large aggregates in and under sea ice and in melt ponds. There is recent evidence from the Arctic that such aggregates can contribute substantially to particle export when sinking from the ice. The role and regulation of microbial aggregation in the highly seasonal, nutrient- and light-limited Arctic sea-ice ecosystem is not well understood. To elucidate the mechanisms controlling the formation and export of algal aggregates from sea ice, we investigated samples taken in late summer 2011 and 2012, during two cruises to the Eurasian Basin of the Central Arctic Ocean. Spherical aggregates densely packed with pennate diatoms, as well as filamentous aggregates formed by *Melosira arctica* showed sign of different stages of degradation and physiological stoichiometries, with carbon to chlorophyll *a* ratios ranging from 110 to 66700, and carbon to nitrogen molar ratios of 8–35 and 9–40, respectively. Sub-ice algal aggregate densities ranged between 1 and 17 aggregates m^−2^, maintaining an estimated net primary production of 0.4–40 mg C m^−2 ^d^−1^, and accounted for 3–80% of total phototrophic biomass and up to 94% of local net primary production. A potential factor controlling the buoyancy of the aggregates was light intensity, regulating photosynthetic oxygen production and the amount of gas bubbles trapped within the mucous matrix, even at low ambient nutrient concentrations. Our data-set was used to evaluate the distribution and importance of Arctic algal aggregates as carbon source for pelagic and benthic communities.

## Introduction

In the Arctic Ocean, sea ice and water column microbial communities both contribute to photosynthetic production, but the relative importance of the pelagic versus the sympagic communities depends on season and geographical region [1]. Depending on light availability, the ice-algal growth season begins in April, and ends in September [2]. The total amount of productivity and standing stock formed seasonally in the water below the ice in the Central Arctic is constrained by light, as well as nutrient availability in the euphotic zone. Annual production in the ice-covered Central Arctic is estimated to be 9–10 g C m^−2^ yr^−1^, which is very low even compared to other oligotrophic oceans [3], [4]. Previous investigations before 1997 have indicated a significant annual contribution by sea-ice algae to total photosynthetic productivity, on the order of 4–57% [3], [5], [6]. The wide range (0–10 g C m^−2^ yr^−1^) of sea ice primary production rates including the Arctic shelves is due to a very high spatial variability [3].

Sub-ice algae can accumulate substantial biomass in the Central Arctic basins, at times exceeding 80% of the standing stock [5]. They offer an additional food source to planktonic grazers in early spring [6] and in late autumn when other food sources are scarce [7], [8]. Also their contribution to carbon export from surface waters can be substantial (1–9 g C m^−2^ yr^−1^) [9], [10]. Ice algae comprise pennate diatoms such as *Nitzschia* sp., *Pseudonitzschia* sp., *Cylindrotheca* sp., *Entomoneis* sp., and *Navicula* sp., which inhabit the ice pores and brine channels of first year (FYI) and multiyear ice (MYI) (e.g., [11]–[13]). The endemic sub-ice diatom, *Melosira arctica*, has a hybrid strategy, growing attached to the underside of ice-floes, of both FYI and MYI, forming filamentous strands of several meters length below the ice [14]–[17]. Different types of large sub-ice algal aggregates (up to 15 cm in diameter) have been observed in the Central Arctic since its first exploration in the 19^th^ century, but due to sampling difficulties still little is known about their physiology, traits and adaptations [18]–[21]. More recently, floating algal aggregations formed mainly by sea-ice pennate diatoms have been observed in melting FYI north of Svalbard and in the Fram Strait [8], [22].

Planktonic diatoms tend to collide and aggregate in nutrient depleted waters after a bloom due to mucus secretion during senescence [23], [24] and in general, sedimenting diatom aggregates contribute significantly to the marine biological carbon pump [25], [26]. Sea-ice algae are known to produce large amounts of extracellular polymeric substances for attachment and cryoprotection [27]–[29]. This may support their aggregation in summer when the ice cracks and melts, and when nutrients become limiting [23]. Algal aggregates eventually sink into deeper water column layers as a consequence of ice break up and melting [9], [15]. Freshly deposited algal aggregates have been observed at the seafloor of the shelves [30] and central deep basins of the Arctic [9], indicating their rapid sedimentation at the end of the summer along the receding ice edge and in the event of substantial under-ice melt.

Many questions remain about aggregate formation, decay, grazing and sinking, as well as their contribution to carbon fluxes in the Central Arctic. Due to the rapid warming of the Arctic leading to a decrease in sea-ice extent and thickness [31], [32] and an increase in the amount of light transmitted through the ice [33], both sea-ice and pelagic phototrophic communities are expected to change with respect to composition and distribution [34], [35], productivity [36], [37], and life cycle [2], [9], [38]. A better understanding of the factors regulating sea-ice productivity, aggregate formation and sinking of sea-ice algae is important for future estimates of the Arctic carbon and nitrogen cycling. By combining field observations with simple experiments, this study examines the relevance of sea-ice algal aggregation for carbon and nitrogen turnover and reservoirs, as well as the processes regulating buoyancy versus sinking. Our data are used to assess the potential importance of algal aggregates for the export of organic matter from the surface ocean to the deep sea.

## Materials and Methods

### Sampling area

**Figure 1:**
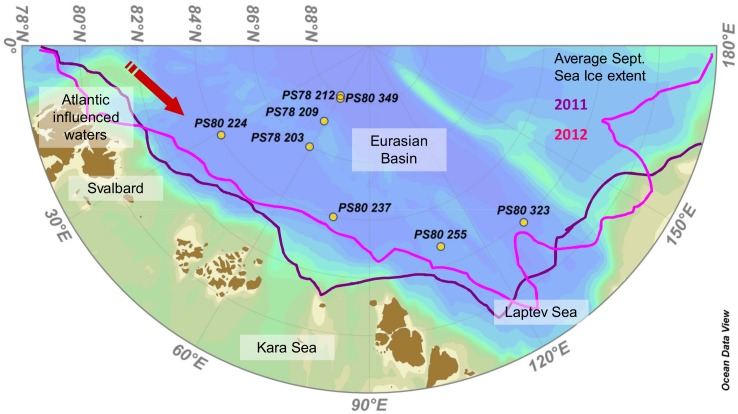
Map of the Arctic Eurasian Basin with sampling stations. Yellow dots represent ice stations where algal aggregates were observed (Expedition PS78/3 took place in summer 2011 and PS80/3 in summer 2012). The purple line corresponds to the September monthly average sea ice extent in 2011 and the pink line in 2012 (Source: http://nsidc.org/data/seaice_index/).

Samples were collected during two cruises to international waters of the Central Arctic Ocean with RV Polarstern in August-September 2011 and 2012 (PS78/3 and PS80/3 respectively) between 81°55′–87°56′N and 31°7′–131°7′E (Fig. 1). Six stations were in international waters and no specific permissions were required for these locations. For the two stations in the Russian exclusive economic zone, the diplomatic permissions for sampling were obtained from the responsible authorities. The field study did not involve endangered or protected species. Data was submitted to PANGAEA (http://doi.pangaea.de/10.1594/PANGAEA.832345).

Replicates of two different algal aggregate types dominated either by pennate diatoms or by *Melosira* were sampled at eight ice stations (Fig. 1). For each station the ice type (MYI or FYI) and the melt pond coverage were assessed [39]. Temperature and salinity of melt ponds containing aggregates were measured *in situ* using a hand-held conductivity meter (315i with TetraCon electrode cell, WTW GmbH, Weilheim in Oberbayern, Germany). Irradiance reaching the aggregates was calculated using the light attenuation coefficients of 1.5 m^−1^ for sea ice, 10 m^−1^ for snow [40] and 0.1 m^−1^ for Atlantic influenced Arctic seawater (based on data from the first expedition, PS78/3), and the daily average total incoming photosynthetically active radiation (PAR) measured with a pyranometer (Kipp&Zonen, Delft, Netherland) mounted on the ship.

### Chemical composition of aggregates

**Figure 2:**
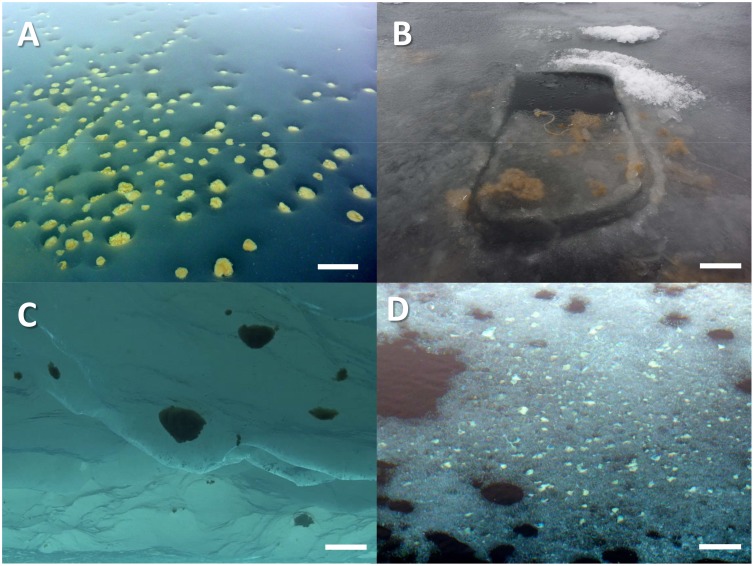
Distribution of aggregates in melt ponds. Degraded pennate aggregates in cryoconite holes at the bottom of a partially open melt pond at station PS78/3_212 (A). *Melosira* filaments hanging from newly formed ice covering an open melt pond at station PS80/3_349 (B). Spherical floating aggregates below sea ice at station PS80/3_237 (Image taken with the ROV Ronja (Courtesy Alfred Wegener Institute Helmholtz Center for Marine and Polar Research (AWI)) (C). Degraded *Melosira* filaments trapped at the bottom of a partially open melt pond at station PS80/3_224 (D). Scale bar = 20 cm.

Several aggregates (between 2 and 20) were sampled at each station together with some ambient water using a manually operated vacuum pump, when found in melt ponds (Fig. 2A), or a plastic ladle, when found in ice cracks. In general aggregates were pooled and homogenized to be able to measure a wide range of biological parameters on standardized subsamples. On two occasions individual aggregates were sampled at stations PS80/3_224 and PS80/3_349 for specific experiments. When classified according to their species composition, pennate diatom aggregates where sampled 6 times and *Melosira arctica* filaments 5 times (Table 1). Aggregate slurry was filtered through a pre-combusted GF/F filter (0.7 µm pore size, Whatman, Kent, United Kingdom) and analyzed with an elemental analyzer (EA3024-IRMS, EuroVetorSpA, Milan, Italy) to determine particulate organic carbon (POC) and particulate organic nitrogen (PON). POC was used to normalize all parameters to carbon mass. For pigment analysis 1–200 ml of the algal slurry (equivalent to 0.1–3 mg C) were filtered through GF/F filters, immediately frozen in liquid nitrogen, and stored at −80°C. Chlorophyll *a* and phaeopigments were measured using high-performance liquid chromatography (HPLC) as described in [41]. The analytical error for the POC, PON and Chl *a* measurements was generally below 2%. For transparent exopolymers (TEP), 1–20 ml of the algal slurry (equivalent to 0.01–2.6 mg C) were filtered in triplicate on to 0.4 µm pore size polycarbonate filters (Nuclepore, Whatman, Kent, United Kingdom), stained with Alcian Blue and stored at −20°C. TEP concentration was measured with the colorimetric method according to [42] and transformed to carbon equivalents as described in [43]. Occasional dilution prior to filtration was accounted for using measured blank values of the dilution water. For dissolved organic carbon (DOC) 10 ml of the algal slurry (equivalent to 0.07–1.3 mg C) were filtered in triplicate through pre-combusted GF/F filters and the filtrate collected in pre-combusted glass vials. The DOC concentration was determined by high temperature catalytic oxidation with a Shimadzu TOC-VCPN analyzer (Shimadzu Scientific Instruments, Kyoto, Japan). Samples were acidified in the auto-sampler and analyzed directly. Nutrients (phosphate, silicate, and nitrate) were measured in seawater, ice and aggregate slurries in an air-conditioned lab container with standard photometric method using a Technicon TRAACS 800 continuous flow auto analyzer (Technicon Corporation) according to established methods [44].

**Table 1:** Algal aggregate types and their degradation stages.

Aggregate type	Pennate spherical		*Melosira* filaments	
Environment	Below ice	Melt Pond	Below ice	Melt Pond
Number of samples	n = 2	n = 4	n = 2	n = 3
Size (cm)	3–15	1–15	1–30	2–15
Abundance (Agg m^−2^)	0.8–5.1	0.1–600	17	9–200
Color	Green-brownish	White-yellowish	Green-brownish	White-yellowish
POC (mg C L^−1^)	24–110	9–86	11–130	7–49
PON (µmol N mg POC^−1^)	8–10	2–8	3–9	2–9
C:N molar	8–11	11–35	9–28	10–40
Chl*a* (µg Chl*a* mg POC^−1^)	5–9	0.01–2	3–5	0.2–1.2
C:Chl*a*	110–210	500–66700	200–340	850–4600
DOC (µmol DOC mg POC^−1^)	7–9	7–11	5–17	7–35
TEP (µg C mg POC^−1^)	0.5–2.5	4–170	2–8	4–17
TEP:POC	0.0005–0.002	0.004–0.17	0.002–0.008	0.004–0.02
NPP (mg C (mg POC)^−1 ^d^−1^)	0.001–0.01	0.001	0.002–0.155	0.002–0.014
Bacteria (cells mg POC^−1^)	3.9E+08–1.5E+09	6.3E+07–6.0E+08	5.6E+08	7.36E+07–6.1E+08

All chemical variables were measured from a homogeneous algal slurry prepared with the aggregates. Units presented are normalized by the POC content of the slurry expressed in mg C per volume of slurry. The carbon normalized NPP represents a carbon turnover estimate for the algae in the aggregate at 50 µmol photons m^−2 ^s^−1^.

### Microscopy of aggregates

The qualitative algal aggregate composition was studied directly on board of the ship with a plankton chamber (Hydro-Bios, Altenholz, Germany) and an inverted light microscope with phase contrast optics (Axiovert 40C, Carl Zeiss, Jena, Germany) and an integrated camera (AxioCamMRc, Carl Zeiss, Jena, Germany). The condition of the diatoms in each sample was defined according to the color and shape of the chloroplasts visible in the interior of the frustules. Green-brownish aggregates contained mostly healthy cells with bright green chloroplasts occupying the entire cytoplasm, while white-yellowish aggregates contained many dead cells with empty frustules or reduced yellowish chloroplasts. To determine bacterial abundance with Acridine Orange direct counts (AODC), sub-samples of the algal slurry were preserved with 2% formalin and sonicated (1×90 s). One milliliter was filtered on to a 0.2 µm pore size polycarbonate filter (Nuclepore, Whatman, Kent, United Kingdom) previously stained with Irgalan black to eliminate autofluorescence [45]. Subsequently the filter was stained with a 0.01% Acridine Orange solution as described in Meyer-Reil (1983) [46]. At least 1000 bacterial cells were counted per filter, using an Axiophot HBO50 (Carl Zeiss, Jena, Germany) microscope. Triplicate filters were counted per sample and the average error was 15%. The percentage of POC contained in the prokaryotic biomass was calculated using the carbon conversion factor of 0.03 pg C per cell specific for Arctic bacteria [47].

### Biomass and primary production in water column, sea ice and aggregates

Although green-brownish sub-ice algal aggregates were observed at all ice stations [9], for logistic reasons we were only able to sample sufficient material at three stations. Here depth-integrated biomass and net primary production (NPP) were calculated for sea ice and water column (PS80/3_224, PS80/3_237 and PS80/3_349) and compared to the integrated sub-ice algal aggregates per m^−2^.

Sea-ice algal biomass was determined by cutting a representative ice core at each station in 10–20 cm slices, melting them in filtered seawater (200 mL of 0.2 µm filtered seawater were added per cm of ice [48]) and filtering them through GFF filters to determine Chl *a* and POC as for the aggregate slurry. For the phytoplankton community in the water below the ice, samples were taken with Niskin bottles disposed in a rosette attached to the Conductivity-Temperature-Depth sensor (CTD) at discrete depths, the water was filtered, and Chl *a* and POC was analyzed as above. Water column phytoplankton biomass was integrated over the euphotic zone (1% incoming irradiance) while sea-ice algal biomass accounted for the ice thickness (Table 2).

**Table 2:** Local areal estimates of fresh sub-ice algal aggregates per aggregate type compared to sea ice and water column at the same location and other studies.

		This Study		Other studies			
Number of stationsLiterature reference		2	1	Glud et al.2014	Assmy et al.2013	Melnikov et al.1997	Gosselin et al.1997
Algal type		Pennate spherical	*Melosira* filaments	Pennate spherical		*Melosira* filaments	
Chl *a* (mg m^−2^)	Sea Ice	1.2–1.7	7.7	0.46±0.29			0.1–14
	Water	2.3–4.3	2.7	59–260			1–445
	Aggregate	0.1–3.7	14–44	2.94±1.21	0.0017–0.0063	22	52–200
	% contribution aggregates	3–38	57–80	0.6–6.5			
POC (mg m^−2^)	Sea Ice	691–896	853				
	Water	1380–1563	1614				
	Aggregate	11–793	3020–9094		0.2–1.3		
	% contribution aggregates	0.5–26	55–78				
PON (mg m ^−2^)	Sea Ice	72–91	95				
	Water	229–231	267				
	Aggregate	1–72	108–324		0.03–0.17		
	% contribution aggregates	0.3–18	23–47				
NPP (mg C m^−2 ^d^−1^)	Sea Ice	1–13	1.5	1.32			57±43
	Water	25–31	1	60–204			30±10
	Aggregate	0.4–9.7	13–40	−3.72	0.002–0.02		included in ice
	% contribution aggregates	1–23	83–94				
Integration parameters	Ice thickness (m)	1.2–1.4	1.4				
	Euphotic zone depth (m)	15–18	12	50			
	Aggregate coverage (%)	0.01–0.03	0.5		0.01–0.03	0.53	
	Aggregate abundance (Agg m^−2^)	0.8–5.1	17				
	Aggregate diameter or length (cm)	3–10	10–30 (length)		0.8–1	40–50 (length)	

Depth-integrated NPP was estimated from ^14^C uptake rates [8], [49]. All aggregate samples were homogenized before spiking the slurry with ^14^C bicarbonate 0.1 µCi mL^−1^ final concentration (NaH^14^CO_3_ solution 52 mCi mmoL^−1^ specific activity, Moravek Biochemicals, Brea, California, USA). Temperature was maintained stable at −1.3°C with a thermo bath (Julabo GmbH, Seelbach, Germany). Three clear bottles with 10 mL each were incubated in the light and one in the dark. The estimated error of the method from the triplicates incubated in the light was 15% on average. Floating aggregate slurries collected at station PS80/3_224 and PS80/3_237 were incubated at 50 µmol photons m^−2 ^s^−1^, which was a typical mid-day *in situ* value. Aggregate slurries from station PS80/3_349, as well as sea ice and water column of all stations were incubated under a range of irradiances (0, 8, 25, 50 and 90 µmol photons m^−2 ^s^−1^) during 24 hours to calculate the *in situ* NPP from photosynthesis vs irradiance curves (PE curves). Using this incubation time NPP is assessed (gross PP minus respiration) [50]. The PE curve was obtained after fitting the data with the equation from [51] using MATLAB. Only regressions with R^2^>0.5 were retained. *In situ* NPP was calculated applying the irradiance measurements at the ice station to the PE curve equation.

To quantify the aggregated biomass and calculate NPP per area, aggregate abundance was quantified from scaled images. At stations PS80/3_224 (Aggregate P4) and PS80/3_237 (Aggregate P5) mean aggregate abundance under the ice was estimated from images taken by the upward looking camera of a Remotely Operated Vehicle (ROV) as described in [8]. At station PS80/3_349, *Melosira* filamentous biomass (Aggregate M5) was estimated from scaled images of the open melt pond (Fig. 2B). The aggregates were photographed with a waterproof camera (Lumix DMC-TS1). The diameter size range used to calculate the aggregate volume, assuming spherical shape, was determined from *in situ* observations of ∼10 aggregates per station before mixing them to form slurry. Thus, the up scaled values in this study represent local estimates for spatial scales of 1–10 m. This approach is different from that of [8] where the mean aggregate diameter was calculated from the ROV dives and used for up scaling to the dimensions of the ice floe. For spherical aggregates, the *in situ* measured diameter size range used in this study was 3–10 cm, while in [8] the mean diameter for the floe scale used was determined to be 0.8–1 cm. For the filamentous aggregates the measured length was 10–30 cm and the observed shape was cylindrical. The minimum and the maximum aggregated biomass per m^2^ was calculated by multiplying the average volume of aggregates (n = 10) by the number of aggregates observed per m^2^ and by the carbon concentration of the aggregate per volume (mg C m^−3^). The aggregate NPP per m^2^ was calculated similarly, by multiplying the total aggregate volume per m^2^ by the measured carbon fixation rate. Chl *a* was estimated from the C:Chl *a* ratio measured in the algal slurry from the same aggregate type. A similar procedure was used for the C:N ratios. Since aggregates showed a very patchy distribution, these calculations reflect local estimates at the sampling spot and cannot be directly up scaled to the Arctic-wide ice-cover.

### Oxygen, carbon and nitrogen turnover of *Melosira arctica* aggregates

On 18 September 2012, station PS80/3_349 (87° 56.01′ N, 61° 13.04′ E), a piece of ice from the frozen surface of an open melt pond was retrieved with algal aggregates attached (Fig. 2B). These were kept in a glass beaker with 800 mL of ambient seawater, at simulated *in situ* conditions: low light (8 µmol photons m^−2 ^s^−1^) and cold temperature (0°C). The initial temperature of the melt pond water collected for the experiment was −1°C. After 12 h in the cold lab-container (0°C) under low light intensity (8 µmol photons m^−2 ^s^−1^), the water reached 0°C and the piece of ice started melting. The vertical position of the aggregates was followed over time and as a function of the salinity gradient and the light intensity, to assess their buoyancy at different environmental conditions. In parallel, sub-samples of the algal slurry from the same type of aggregate were incubated under different conditions to assess oxygen, carbon and nitrogen fluxes.

Five sub-samples were incubated under different light intensities to estimate NPP from ^14^C uptake rates, as described above. In addition, oxygen concentration was followed in the same vials using fiber optic oxygen sensors (FireStingO2, PyroScience GmbH, Aachen, Germany). Oxygen sensitive sensor spots were glued to the interior of the glass vials previous to the experiment. The sensor spots contain an oxygen quenchable fluorophore (oxygen sensitive dye) which changes its fluorescence properties according to the oxygen concentration [52]. After a two point calibration at the temperature of the experiment, in air saturated and oxygen free seawater, oxygen concentration was measured in each vial prior to the experiment, after 2, 7, 17 and 28 h of incubation. NPP was calculated from the slope between oxygen concentration at each time point (regression R^2^>0.93) and a photosynthetic quotient of 1.25 was used to convert the oxygen exchange to carbon equivalents [53].

To measure the nitrate uptake, a 50 mL slurry sub-sample was incubated at 50 µmol photons m^−2 ^s^−1^ and nitrate was measured initially and after 1 and 4 days. In parallel, 18 subsamples of the algal slurry were incubated in 12 mL exetainers (Labco Inc., Buckinghamshire, England) to measure the denitrification and nitrification potential. Subsamples of the slurries were spiked either with ^15^NO_3_, with ^15^NH_4_^+^ or with a mixture of ^14^NO_3_ and ^15^NH_4_^+^ (final concentration 50 µmol L^−1^), using a modified version of the method described in [54]. One sub-sample of each treatment was placed at 50 µmol photons m^−2 ^s^−1^, while the remaining sub-samples were placed in darkness. The gradual O_2_ consumption was followed in both set ups using the optode system described above. Anoxic conditions were reached after two days in the dark. The first time series was ended immediately by injecting 100 µL ZnCl_2_ into the exetainers, while the remaining samples were terminated as the oxygen level reached 67, 35, and 0%, after 0.8, 1.2 and 2 days respectively. The isotopic composition of the N_2_ was measured using a gas chromatograph coupled to an isotope ratio mass spectrometer through a ConFlo-III interphase [55]. Calculations for denitrification rates were done according to [56]. The remaining sample volume was subsequently filtered through disposable, 0.45 µm filters and concentrations of ammonium, nitrate and nitrite (NO_x_) were determined [57], [58].

## Results

### Sea ice and melt pond observations

During August and September 2011 and 2012, FYI in a late melting stage was the dominant ice type at seven of the eight ice stations investigated (Table S1 and Table S2). Sea-ice thickness was between 0.7 and 2 m and melt pond coverage ranged between 10 and 50%. Only one MYI floe could be sampled during both cruises. The melt ponds sampled along the eight ice stations, had different depths (0.3–1 m), salinities (0–32), were open or closed to the seawater below them, and varied in ice cover. In partially open melt ponds at stations PS78/3_212 (Fig. 2A) and PS80/3_224 (Fig. 2D), a steep salinity gradient with 0 at the surface and values of 28–30 at the bottom (∼0.4 m depth) was measured. Closed melt ponds at stations PS78_203, PS78/3_209, PS80/3_224 and PS80/3_255 had a steep salinity gradient too, but the maximum salinity at the bottom (0.3–0.6 m depth) was generally lower than in open melt ponds (Table S1 and S2). Aggregates were located either at the bottom of the melt pond where the salinity was highest or frozen within the ice cover (Fig. 2B).

### Types of algal aggregates and their degradation stages

**Figure 3:**
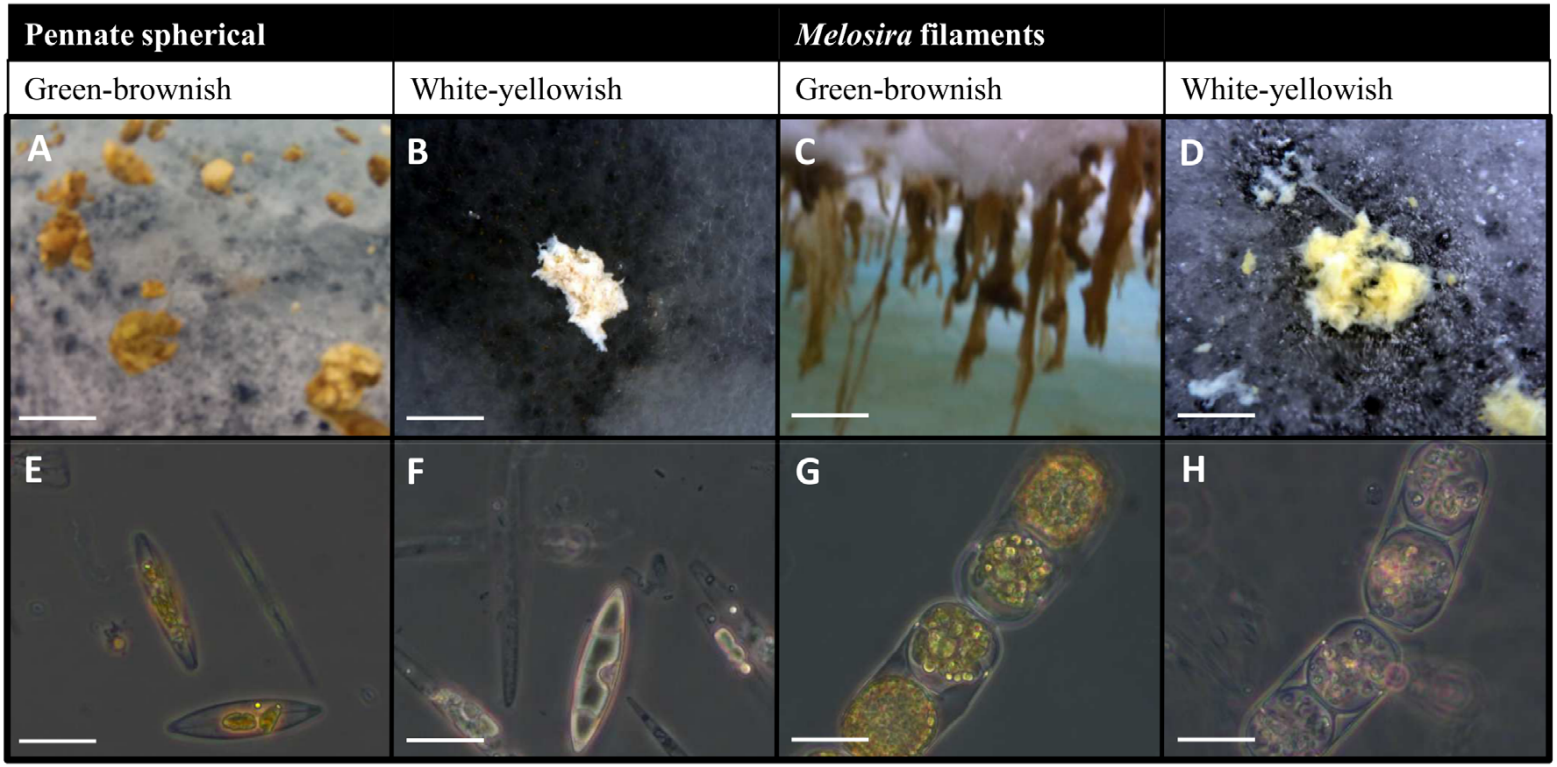
Representative types of aggregates observed. Macroscopic images of the aggregates (A–D) and their microscopic composition (E–H). Fresh spherical floating aggregates below the ice formed by pennate diatoms (A and E). Degraded aggregate trapped in a closed melt pond formed by dead pennate diatoms with empty frustules (B and F). Filaments hanging from newly formed ice over an open melt pond formed by *Melosira arctica* cells that contain green chloroplasts (C and G). Degraded aggregate formed mainly by dead *Melosira arctica* cells trapped at the bottom of a partially open melt pond (D and H). Scale bar = 5 cm (A), 2 cm (B), 10 cm (C and D), and 20 µm (E–H).

By macroscopic and microscopic observations of the diatom composition (Fig. 3), two different types of algal aggregates were identified: (1) spherical aggregates floating under the ice or trapped in melt ponds, mainly composed of pennate diatoms (*Nitzschia* sp., *Navicula* sp., *Fragilariopsis* sp., and *Entomoneis* sp.) (Table S1), and (2) hanging filamentous strings, mainly composed of the centric diatom *Melosira arctica* (Table S2).

Both the pennate and centric diatom aggregates were present in different degradation stages (Fig. 3). Their characteristics are summarized in Table 1. Fresh, green-brownish aggregates of both types were found below the sea ice, either floating or attached to the ice (Fig. 2B), while degraded, yellow-whitish aggregates were usually found at the bottom of melt ponds in cryoconite holes (Fig. 2A). Microscopic observations confirmed that the green-brownish aggregates were formed by diatoms with healthy-looking chloroplasts in their cytoplasm (bright green under the light microscope and occupying almost the entire cytoplasm), while the yellow and white aggregates contained a high proportion of empty frustules (Fig. 2). Green-brownish aggregates generally occurred where nutrient concentrations were above 2 µmol L^−1^ nitrate, 0.2 µmol L^−1^ phosphate, and 2 µmol L^−1^ silicate at the bottom of melt ponds or below the ice.

White-yellowish aggregates had higher C:N molar and C:Chl *a* mass ratios than green-brownish aggregates (Table 1). The highest Chl *a* values were encountered in dark green-brown aggregates (Fig. 3A), and the lowest values were observed in aggregates with white coloration (Fig. 3B). The stickiness of the aggregates is reflected in the TEP:POC ratio (0.001–0.17), that was highest in aggregates found in melt ponds where the nutrients were low (nitrate <0.2 µmol L^−1^, phosphate <0.02 µmol L^−1^and silicate <2 µmol L^−1^) (M1 and M4, Table S2). In general, degraded aggregates contained more TEP than fresh aggregates (Table 1).

Bacterial abundance in the aggregate slurries spanned two orders of magnitude, from 6.3×10^7^ to 1.5×10^9^ cells mg POC^−1^, making up 0.2–20% (Median of 1.2%) of the POC. Greenish-brownish aggregates hosted one order of magnitude more bacteria than white-yellowish aggregates. The DOC concentrations of the algal aggregates ranged between 5 and 35 µmol DOC mg POC^−1^ (Table 1), making up 4–30% of the total carbon (sum of POC and DOC). This corresponds to an average of 295 µmol L^−1^ DOC in the algal slurries, which is higher than the average DOC concentration in Central Arctic surface waters (58 µmol L^−1^ DOC [59]). White-yellowish *Melosira* filaments had slightly higher DOC concentrations than green-brownish filaments, although the difference was not statistically significant. Even though no quantification of grazing rates was performed, the observation of active ciliates, particularly in degraded aggregates and in some fresh aggregates, suggests that aggregates served as food source for sympagic meiofauna. In addition, grazing copepods and amphipods were observed in some spherical pennate aggregates. However, no such grazers were observed on green-brownish *Melosira arctica* filaments.

### Contribution of algal aggregates to system scale phototrophic biomass and primary production

Due to their patchy distribution and inaccessibility, green-brownish sub-ice aggregates could only be sampled at three stations although they were observed with the ROV at all eight stations investigated. Yellow-whitish aggregates reached highest abundances in melt ponds (stations PS78/3_212 (Fig. 2A) and PS80/3_224 (Fig. 2D)).

Table 2 compares the integrated phototrophic biomass of sea ice (1.2–1.4 m thickness), water column euphotic zone (12–18 m depth), and the two different types of aggregates investigated, including previous observations on pennate diatom [8], [22] and *Melosira* based aggregates [5], [14]. Under-ice ROV surveys revealed a high degree of horizontal patchiness in aggregate abundance below the ice (Fig. 2C). Locally, spherical floating aggregates reached an abundance of 0.8–5.1 aggregates m^−2^ and a diameter of 3–10 cm, containing 0.1–3.7 mg Chl *a* m^−2^, which corresponded to 3–38% of total phototrophic biomass. *Melosira arctica* filaments observed in autumn 2012 were 10–30 cm long and reached abundances of 17 aggregates m^−2^ with 14–44 mg Chl *a* m^−2^, corresponding to 57–80% of total phototrophic biomass.

At the sampled sites, floating aggregates contributed with 0.5–26% (pennate), and 55–78% (*Melosira)* to the particulate organic carbon pool of the integrated euphotic zone (Table 2). Pennate-diatom aggregates showed Chl *a*/CPE values similar to the sea-ice algae at the same station, while *Melosira* filaments showed Chl *a*/CPE values similar to the pelagic communities. In general the C:N ratios of green-brownish aggregates (8–28) were higher than the values for the phytoplankton (∼5) and the sea-ice algae (6–8). Green-brownish *Melosira* aggregates showed higher C:N ratios (9–28) than all pennate aggregates (8–11). In white-yellowish aggregates C:N ratios were generally higher (11–35 in pennate and 10–40 in *Melosira* aggregates).

Regarding *in situ* NPP per m^2^, spherical floating aggregates showed similar NPP as the corresponding integrated value for sea ice, and 50% of the value for the depth-integrated water columns euphotic zone (15–18 m) (Table 2). In contrast, up scaled *Melosira arctica* filaments reached NPP rates one order of magnitude higher than the values of the integrated water columns euphotic zone (12 m) and the sea ice in late September (Table 2). The daily average irradiance received below the ice *in situ* at stations PS80/3_224 and 237, where pennate aggregates were found, was 99 and 52 µmol photons m^−2 ^s^−1^, respectively. Hence, *in situ* NPP of aggregates at station 224 may be underestimated, since the incubation performed at 50 µmol photons m^−2 ^s^−1^.

**Figure 4:**
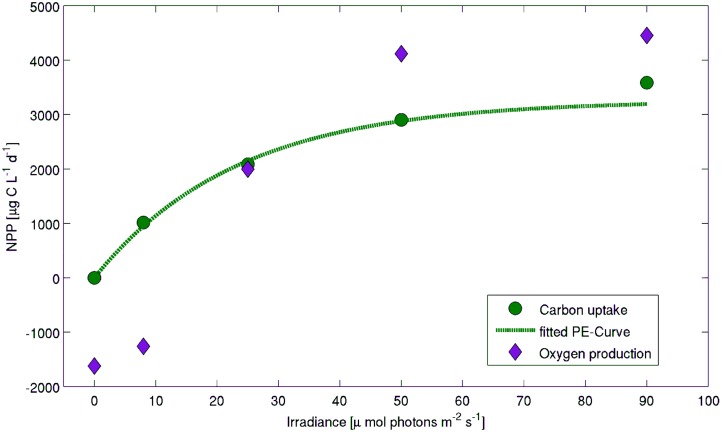
Photosynthesis vs Irradiance curve of *Melosira arctica* algal slurry (M5). Carbon uptake measured with the ^14^C radioactive isotope method (green circles) and oxygen production or consumption using optodes (purple diamonds) of the algal aggregate slurry from the *Melosira arctica* aggregate used for the buoyancy experiment (Fig. 5). The threshold irradiance for oxygen production is around 20 µmol photons m^−2 ^s^−1^. Oxygen rates have been transformed to carbon equivalents using the photosynthetic quotient of 1.25 to improve the visualization of the two different methods used to measure NPP.

For the *Melosira arctica* filaments found at station PS80/3_349, both carbon uptake and oxygen production were monitored in homogenized algal slurries at different light intensities. The fitted PE curve of the carbon uptake (R^2^ = 0.986), showed a photosynthetic maximum (P_max_) of 3.7 mg C L^−1 ^d^−1^, an initial slope of 120 µg C L^−1 ^d^−1^ (µmol photons m^−2 ^s^−1^)^−1^ and no photoinhibition at irradiances lower than 90 µmol photons m^−2 ^s^−1^ (Fig. 4). Carbon uptake could be quantified even at the lowest irradiance (8 µmol photons m^−2 ^s^−1^). However, no net oxygen production took place at this irradiance, rather oxygen was consumed (Fig. 4). At light levels above 20 µmol photons m^−2 ^s^−1^, oxygen production was detected.

### Buoyancy test

**Figure 5:**
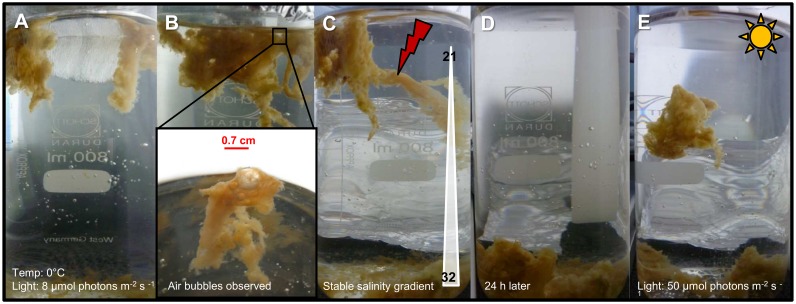
Buoyancy test. (A) A piece of ice with several attached filamentous algal aggregates was kept in a glass beaker at simulated *in situ* conditions. (B) Once the ice disappeared it was observed that air bubbles were trapped in the mucous matrix of the floating aggregates. (C) Despite the salinity gradient, one of the aggregates sank when the air bubbles were removed. (D) 24 h later all algal aggregates had sunk. (E) Only when increasing the light intensity, some of the algal aggregates were able to produce enough oxygen to regain buoyancy. Scale bar = 2 cm.

Besides the observations of floating aggregates in the laboratory, some aggregates were observed floating below the ice, others at the pycnocline in open melt ponds and also coming up to the surface through bore holes in the ice. To investigate the causes of buoyancy, a simple test was performed with intact aggregates (n = 4) anchored to newly formed ice from station PS80/3_349, composed of a mixture of *Melosira arctica*, *Cylindrotheca* sp., and *Nitzschia* sp. (Fig. 3C and G, Fig. 5A) (M5 in Table S2). Once the ice had melted, the four intact algal aggregates remained floating at the surface (Fig. 5B). It was then observed that air bubbles of different sizes (0.01 to 0.7 cm) were trapped in the mucous matrix of the floating aggregates (Fig. 5B bottom). To test if these air bubbles were responsible for the aggregates buoyancy, the bubbles of one aggregate were physically removed. The aggregate devoid of bubbles sunk to the bottom of the beaker, regardless of the stable salinity gradient formed due to the ice melt (Fig. 5C). After 24 hours, the remaining aggregates also sank to the bottom of the beaker (Fig. 5D). To test if photosynthetically produced oxygen was the source of the aggregate bubbles, we increased the light intensity reaching the sunken aggregates in the beaker. After increasing the irradiance to 50 µmol photons m^−2 ^s^−1^ for 48 h, the sunken aggregates formed bubbles and regained their buoyancy (Fig. 5E), indicating that photosynthetically produced oxygen might be responsible for the gas bubble formation.

### Oxygen and carbon turnover in algal aggregates

The *Melosira* slurry showed a bulk oxygen consumption rate of 0.13±0.02 mmol O_2 _L^−1 ^d^−1^ at low irradiance (8 µmol photons m^−2 ^s^−1^). Non-invasive measurements with microsensors on one pennate-diatom also showed net oxygen consumption with almost anoxic conditions (10–120 µmol L^−1^ Oxygen) in the center of the aggregate (see Fig. S1).

Assuming a 1∶1.25 ratio of CO_2_ production to O_2_ respiration we transformed the oxygen consumption rate mentioned above into carbon respiration rate. Dividing the POC measured in each sample (Table S2) by the carbon respiration rate, we estimated the carbon turnover in the *Melosira* slurry. The turnover of particulate carbon at low irradiance (8 µmol photons m^−2 ^s^−1^) would be 8–11 days. Net oxygen consumption and carbon remineralization occurred at lower light intensities in the *Melosira* algal slurry than in the compact pennate-diatom aggregate. Floating green-brownish aggregates incubated for 4–5 days at low irradiances lost their buoyancy and became net heterotrophic. Oxygen production in both aggregate types was probably light regulated. However, this observation is based on single measurements, with the two respective types of aggregates. Further experiments are needed to fully conclude that light-regulated oxygen production is responsible for aggregate buoyancy.

### Nitrogen cycling in *Melosira* aggregates

Nitrate and ammonium concentrations were monitored in parallel in the *Melosira* algal slurry sample incubated under *in situ* representative light conditions (50 µmol photons m^−2 ^s^−1^). During the first 24 h, the nitrate consumption rate was 0.94 µmol N L^−1^ and the ammonium consumption 2.47 µmol N L^−1^. Denitrification and nitrification potential was measured in slurry samples spiked with labeled nitrate and ammonium, respectively (^15^NO_3_ and ^15^NH_4_^+^). During light, nitrate was produced at a rate of 1.7±0.001 µmol L^−1 ^d^−1^ and ammonium was consumed at a rate of 1.6±0.003 µmol L ^−1 ^d^−1^. In the dark, nitrate was produced at lower rates (0.4–0.5 µmol L^−1 ^d^−1^) and high net ammonification rates were measured (1.5–5.8 µmol L^−1 ^d^−1^). A potential for denitrification could be detected (2–5 nmol L^−1 ^d^−1^) under anoxic conditions. Aggregates appeared to be hot spots for nitrogen cycling with potential for ammonification, nitrification and denitrification, and the rates seemed to be regulated by ambient light availability.

## Discussion

### Aggregate formation, distribution and degradation

Different types of aggregations of algae have been described in Arctic sea ice, mostly by observations made during summer, below pack ice [8], [14], [15], [21], [22]. At the end of the productive season, floating sub-ice algal aggregates tend to accumulate in dom-shape structures below the ice or in half open melts ponds or cracks. They have a very patchy distribution that seems to be governed by ice topography.

**Figure 6:**
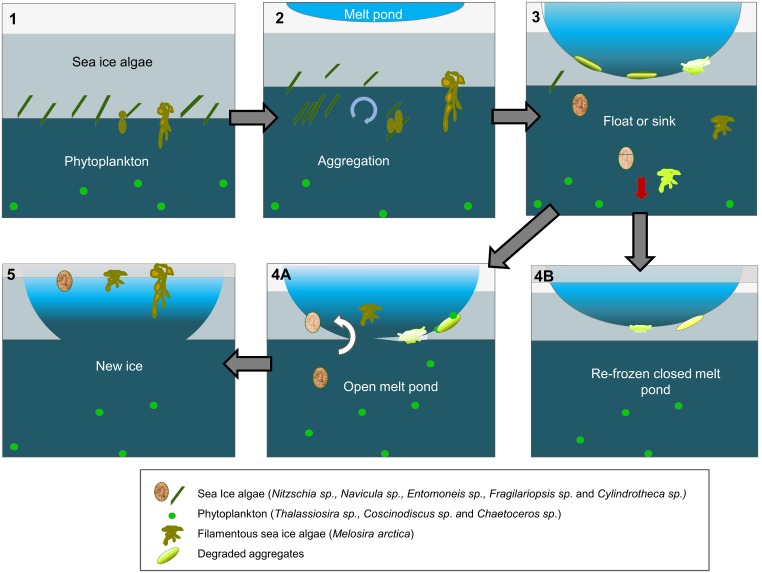
Conceptual model of the mechanisms responsible for the formation and fate of the different types of algal aggregates. (1) In early spring sea-ice algal growth starts before that of phytoplankton. (2) In summer as the sea ice melts and nutrients become limiting, some sea-ice algae are released to the water column or grow into the water column. Due to their stickiness and the under-ice turbulence they form aggregates. (3) In late summer melt ponds grow in depth and the sea-ice algae that are still in the ice are gradually exposed to very low salinities, high irradiances, and nutrient depletion making them accumulate and degrade in the pond. Depending on the environmental conditions, some sub-ice aggregates sink and others remain floating. (4) In early autumn, the melt ponds can either open completely allowing some phytoplankton species to come into the melt pond, or they can refreeze again, becoming second year ice. (5) In autumn those melt ponds that were open to the seawater freeze again, trapping the floating aggregates that were not grazed in the newly formed ice.

Based on our measurements and a synthesis of previous studies, we propose a conceptual model for aggregate formation and degradation in the Arctic (Fig. 6). This concept is based on the current trends of thinning sea-ice and higher melt pond coverage [60], [61], but it only applies to the current situation and not to the Arctic ecosystem of three decades ago and it remains unclear if and for how long it can be projected into the future. Considering an average ice thickness of 1–2 m, light for photosynthesis is available to ice algae already in April-May, and single cells of sea-ice algae that have survived the winter darkness [62], [63] can grow in the brine channels of the ice matrix [3] (Fig. 6.1). In summer, ice melting from the top and the bottom releases diatoms into ice cracks, melt ponds and the water column, respectively [8], [21], [22] (Fig. 6.2). Since sea-ice diatoms produce high concentrations of transparent exopolymers in the ice [27], [64] they tend to aggregate under moderate turbulence and shear, creating algal flocs [65], [66].

Newly formed melt ponds are usually shallow, light blue, have low salinity, and contain little visible life [67], [68]. As melt ponds grow in depth during summer, the freshwater pool increases and sea-ice algae living at the bottom of the ponds or in pond water are gradually exposed to higher irradiances, lower salinity, and less nutrients (Fig. 6.3). Since sea-ice algae are adapted to low light [69], [70], low temperature and high salinity [48], [71], these new conditions could trigger the exudation of polysaccharides that create a mucous matrix around the cells [72], thereby increasing their stickiness and their predisposition to aggregation. This is probably the case for pennate diatom species growing in the ice as single cells and aggregating after their release during ice melt. However, the centric diatom *Melosira arctica*, grows forming chains and excretes high amounts of mucus that contribute to the formation of filament-shaped aggregates [73]. Nutrient depletion can also increase diatom exopolymer exudation [23], [28], [74], contributing to aggregation. In autumn 2011, we observed many previous ponds, which had opened to the underlying seawater, allowing an exchange of phytoplankton species between seawater and the sea ice habitat (Fig. 6.4A), for example at station PS78/3_212 (Fig. 2A, Table S1). Around mid-September ice-melt stops and melt ponds close again when their surface and bottom refreezes (Fig. 6.4B). Mostly white-yellowish, apparently degraded aggregates were found in this type of closed melt pond in late autumn (Fig. 3B). Some diatoms, such as *Melosira arctica*, build up dense biomass accumulations despite nutrient constrains, that can be exported to the deep sea upon rapid melting of the sea ice [9], [75]. Probably the attachment to the lower part of the ice enables them to harvest nutrients from a wider area while drifting over large distances. Some of them can maintain buoyancy throughout summer and can then be refrozen into the newly formed ice in early autumn (Fig. 6.5) [68].

### Relevance of sea-ice algal aggregates for Arctic carbon and nitrogen fluxes

The spatial and temporal patchiness remains a challenge when estimating the importance of algal aggregates for ecosystem productivity and carbon flux [8], [30]. Assmy et al., 2013 measured rather small Chl *a* concentrations (2–6 µg Chl *a* m^−2^) when up scaling the contribution of the observed aggregates to the area of the entire ice floe. Here we assessed the standing stock and productivity at a local scale, for two different types of sea-ice algal aggregates. Our study took place at the end of the summer, when nutrient depletion limits pelagic productivity. During this period, we found that algal aggregates contributed significantly to total biomass at a local scale in and below the sea-ice.

The estimates of Chl *a* reported here for spherical aggregates formed by pennate diatoms (Table 2) is within the range of values reported earlier (2.9±1.2 mg Chl *a* m^−2^; Fram Strait, [22]). Locally, higher Chl *a* values were reached by *Melosira arctica* filamentous aggregates in late September, contributing 57–80% to total phototrophic biomass when phytoplankton activity is reduced in the water column (Table 2). Also these values fall in the range of values previously reported for this type of *Melosira* sub-ice assemblage associated with MYI (22 mg Chl *a* m^−2^ [14]) or FYI (52–200 mg Chl *a* m^−2^ [5]). This suggests that ice algal aggregations could be a relevant food source in a wider area partly for under-ice zooplankton and for nekton at the end of the season and potentially also in winter, before the aggregates freeze into the ice [8], [76], [77]. In contrast, for *Melosira* aggregates, grazing has only rarely been observed [13] and much of their biomass may sink out earlier in the season [9], [15], [30]. Observations of regionally widespread algal falls to the seafloor indicate that algal aggregates are not only locally relevant as hot spots of microbial activity, but may also contribute substantially to total carbon export [9].

Similar conclusions as to the relative contribution to biomass are reached using the POC values of the aggregates compared to the total sympagic or pelagic community. The contribution to total PON at a local scale was lower (0.3–18%) for pennate aggregates than for *Melosira* filaments (23–47%). The overall relatively low mass ratios of C:Chl *a* of 110 and 212 for green-brownish pennate and centric diatom aggregates, respectively, indicate a healthy algal community. However, compared to the water column C:N ratio of 5–6, the green-brownish aggregates had an elevated C:N ratio (8–11 and 9–28 for green-brownish pennate and centric diatom aggregates, respectively). The white-yellowish aggregates containing many empty frustules even reached ratios of 40. This suggests an important contribution of exudate-carbon to the aggregates.

Pennate aggregates can contribute 1–23% to total *in situ* integrated NPP at a local scale, and *Melosira arctica* filaments as much as 83–94% of the total NPP north of 80°N in mid-September, when very little production is taking place in the water column or in the sea-ice. *Melosira* filaments can be more productive than the integrated euphotic zone in and below the ice in the Central Arctic (Table 2). However, aggregate slurries may overestimate NPP, due to the reduction of light adsorption by dilution compared to the naturally densely packed aggregates, hence the total contribution of intact aggregates is likely lower [22]. Indeed, intact spherical aggregates incubated for some days in the laboratory under low light conditions appear to be net heterotrophic (Fig. S1).

Another aspect of the contribution of sea-ice algal aggregates to carbon fluxes in the Arctic Ocean is the contribution to particle export. Diatom aggregates can substantially enhance carbon flux from the pelagic realm to the benthos [24]–[26]. In the Arctic, sea-ice algae are main contributors of the vertical export flux in summer [10], [78]. In 2012 the direct export of sea-ice algal aggregates was observed in relation to sea ice melt [9]. It was estimated that their contribution to the total carbon flux could reach 85% and from our results we can estimate that their contribution to the particulate nitrogen sinking flux potentially would be around 47% of the PON present at the end of the productive season. Considering the already low nutrient concentrations of the Central Arctic surface waters, such an export could only be sustained on decadal scales, if more nutrients would be mixed in, e.g. by increasing wind mixing, or by increasing transport from the Arctic shelves [79]. Due to their relevance at least for standing stock and export flux, it is important to better understand the mechanisms of sea-ice aggregate formation, buoyancy regulation and sedimentation.

### Buoyancy regulation

Adaptations of phytoplankton cells to control buoyancy include the formation of gas vacuoles, lipid accumulation, ion exchange and morphological features like hairs and spines [24], [80], [81]. In the Arctic Ocean, the observation of algal aggregates floating below the pycnocline was previously explained by accumulation at vertical density gradients [8], [19], [21]. However, in our experiment the aggregates sank after physical removal of air bubbles trapped in the mucous matrix (Fig. 5). The salinity gradients might determine where aggregates accumulate, but density gradients were not the buoyancy regulating mechanisms *per se*. Both pennate and centric diatom-formed aggregates studied here showed gas bubbles trapped in the aggregates mucous matrix, which seem to be a relevant mechanism for aggregates to stay afloat. Previous physiological studies with another centric diatom have shown a correlation between increased light input and reduced sinking rates [81]. The results from our experiment that the sunken aggregates can regain buoyancy in situations favorable for photosynthesis, i.e. by increased light intensity, suggests that the balance between oxygen production by photosynthesis and consumption by respiration was the key factor for buoyancy regulation in the aggregates we investigated. Since aggregates are not closed systems, the rate of oxygen production plus the impeded diffusion across the mucus matrix of the aggregate minus oxygen consumption by the sea-ice algae themselves, bacteria and zooplankton, will determine the amount of gas available to maintain buoyancy [22]. However, further experiments are needed to confirm this hypothesis.

Other factors affecting buoyancy are the density of the aggregate. Diatoms exudates have been observed to be positively buoyant and to facilitate the attachment of bacteria to the aggregate [82]. Therefore, TEP do not only play an important role in aggregation but could also be important in buoyancy regulation. The TEP:POC ratios presented in this study (0.001–0.2) were generally lower than the ones reported for land fast sea ice in spring (0.08–0.72) [82], but in the same range reported for other marine diatom aggregates (∼0.25; [83]) and higher than the TEP:POC ratios measured in the water column surrounding the aggregates.

### Fate of the sea ice algal aggregates

Detrimental conditions to the aggregate community such as very low salinity from melt water, seawater warming, high grazing pressure, or nutrient depletion will eventually lead to buoyancy loss, degradation and sinking [9], [15]. This could result in their accumulation at the bottom of melt ponds, or export to the sea floor [9], [30]. White-yellowish aggregates of high TEP:POC, C:Chl *a* and C:N ratios indicating substantial degradation of algal cells [43] were often found trapped in melt ponds where nutrients were depleted. Green-brownish aggregates contained more bacteria than white-yellowish, degraded aggregates, following the general correlation between bacterial abundance and Chl *a* observed in the ocean [84], suggesting that labile carbon supplies from the algae to the bacteria are still present in green-brownish aggregates at the end of the season. Despite the fact that bacterial abundances per carbon weight were in the lower range of values published for Antarctic sea-ice [82], they were still two orders of magnitude higher (10^7^–10^9^ cells mg POC^−1^) than in the ambient water (10^5^–10^7^ cells mg POC^−1^) suggesting that aggregates are a rich substrate for bacteria. The higher concentrations of DOC in the aggregates compared to the average DOC concentrations of Arctic surface seawater [59] indicate that algal aggregates might be a source of DOC, in which carbon is rapidly remineralized.

Net heterotrophic conditions were measured in floating sea-ice algal aggregates during summer indicating a rapid decomposition of the algal biomass in the aggregates ([22] and this study, also see Figure S1, Table S1, and Table S2). Moreover, observations of photosynthetically active diatom cells in aggregates exported to the deep-sea [9] and subsequent oxygen depletion in the seafloor suggest rapid sedimentation and microbial remineralization of the aggregates. Nitrate production matched the ammonium consumption in the *Melosira* aggregate slurry, suggesting that nitrification might take place as well under photosynthetic conditions at high light intensities. This means that decaying algal aggregates could be sources of nitrate to the surrounding seawater in an otherwise nitrogen-limited system. Previously it has been speculated that internal nutrient regeneration by bacterial remineralization inside aggregates can support growth of at least a fraction of the algal population [85], [86]. Denitrification, revealed by N_2_ production, may occur in the anoxic center of intact aggregates [87], but the potentials encountered in *Melosira* algal slurry were low (0.002–0.005 µmol L^−1 ^d ^−1^). This could indicate that the aggregates might remove considerable amounts of carbon and nitrogen from surface waters by export. Using the aggregate carbon export of 9 g C m^−2^ estimated previously for 2012 (Boetius et al. 2013), and the median C:N ratio of 10 of the fresh aggregates (n = 4), approximately 45% of carbon fixed as new production since last winter and 36% of surface nitrogen consumed by sea-ice algae and surface water phytoplankton was exported to depths >4000 m.

In conclusion, we suggest that pennate diatom and *Melosira arctica*-based ice-algal aggregates contribute substantially to nutrient and carbon cycling and export in the Arctic. Preliminary experiments suggest that their buoyancy is regulated by photosynthetically-produced oxygen. With the current trend of warming of the Arctic causing sea ice retreat as well as thinning of the ice cover that increases drift speed, it is likely that sea-ice algae can grow faster and earlier in the season harvesting nutrients from a wider area of surface waters, but will more often meet unfavorable conditions during summer, e.g. nutrient limitation, freshening and melting of their habitat favoring aggregate formation and degradation. The advantages of the formation of aggregates compared to the single-cell or short-chain life style of pelagic sea ice algae is uncertain. Aggregation into large clumps and filaments may protect diatom species such as *Melosira arctica* from high irradiances. Trapping of gas bubbles in the aggregates enhancing floatation and eventually serving entrapment into the ice in late autumn might be another advantage. The intense nitrogen cycling observed in the aggregates indicates that they could play a role in nutrient cycling in the oligotrophic Arctic Ocean. It remains methodologically difficult, but important for a better understanding of carbon and nutrient budgets as well as for the ecology of the Arctic Ocean, to quantify temporal and regional variations in aggregate formation, distribution and fate on the ecosystem scale.

## Supporting Information

Figure S1 **Oxygen profile inside degrading pennate diatom aggregate (P5).** Oxygen microprofiles (n = 3) measured using an oxygen microoptode (FireStingO2, PyroScience GmbH, Aachen, Germany) in a 5 cm diameter spherical pennate-diatom aggregate incubated in a beaker in the lab for 3 days at 50 µmol photons m^−2 ^s^−1^ and −1.3°C. Oxygen microprofiles across the water-aggregate interface were measured with steps of 0.5 mm and since the original spherical diatom aggregate started to flatten the diffusive oxygen uptake (DOU, mmol m^−2 ^d^−1^) was calculated using Fick’s first law of diffusion DOU = D_0_ (dC/dz), where D_0_ (cm^−2 ^s^−1^) is the molecular diffusion coefficient in water, C (µmol L^−1^) is the solute concentration, and z (cm) is the depth within the aggregate. The total oxygen consumption rate of the aggregate was calculated integrating the diffusive flux over the entire aggregate surface area [88]. Using a typical pennate-diatom aggregate size of 5 cm in diameter and a spherical shape (Volume of one aggregate = 0.06 L) the corresponding O_2_ consumption rate of a degrading pennate aggregate is 1.8±0.2 mmol O_2 _L^−1 ^d^−1^ (n = 3).
(TIFF)

Table S1 **Characteristics of pennate algal aggregates and sea ice stations investigated.**
(DOCX)

Table S2 **Characteristics of Melosira algal aggregates and sea ice stations investigated.**
(DOCX)
