# Supplementary figures and images for: Composition, Buoyancy Regulation and Fate of Ice Algal Aggregates in the Central Arctic Ocean
Source: PLoS One. 2014 Sep 10;9(9):e107452. doi: 10.1371/journal.pone.0107452 (PMC4160247; doi:10.1371/journal.pone.0107452)

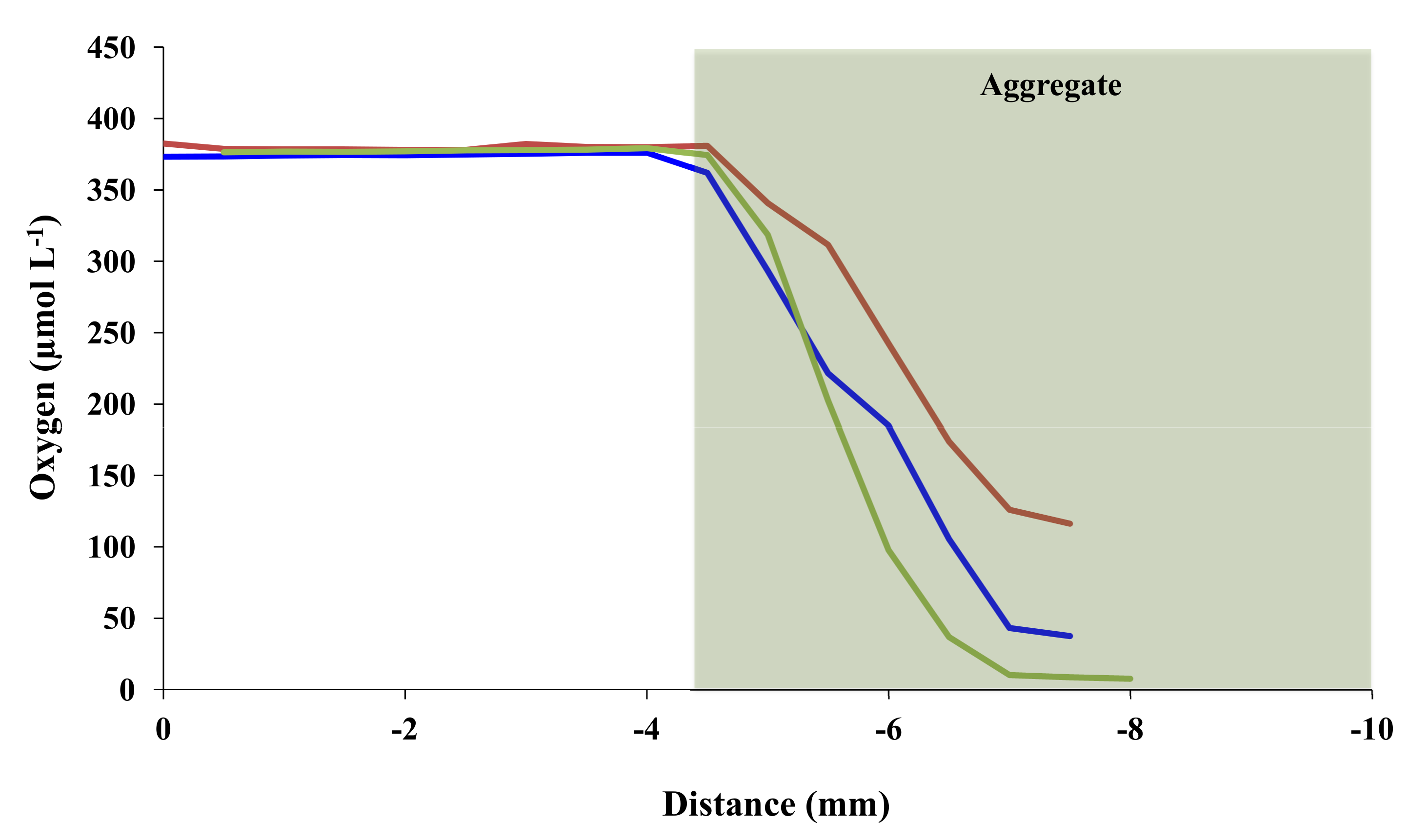

Supplement: Figure S1 — Oxygen profile inside degrading pennate diatom aggregate (P5). Oxygen microprofiles (n = 3) measured using an oxygen microoptode (FireStingO2, PyroScience GmbH, Aachen, Germany) in a 5 cm diameter spherical pennate-diatom aggregate incubated in a beaker in the lab for 3 days at 50 µmol photons m−2 s−1 and −1.3°C. Oxygen microprofiles across the water-aggregate interface were measured with steps of 0.5 mm and since the original spherical diatom aggregate started to flatten the diffusive oxygen uptake (DOU, mmol m−2 d−1) was calculated using Fick’s first law of diffusion DOU = D0 (dC/dz), where D0 (cm−2 s−1) is the molecular diffusion coefficient in water, C (µmol L−1) is the solute concentration, and z (cm) is the depth within the aggregate. The total oxygen consumption rate of the aggregate was calculated integrating the diffusive flux over the entire aggregate surface area [88]. Using a typical pennate-diatom aggregate size of 5 cm in diameter and a spherical shape (Volume of one aggregate = 0.06 L) the corresponding O2 consumption rate of a degrading pennate aggregate is 1.8±0.2 mmol O2 L−1 d−1 (n = 3). (TIFF) [file pone.0107452.s001.tiff]
