# Supplementary material for: Composition, Buoyancy Regulation and Fate of Ice Algal Aggregates in the Central Arctic Ocean
Source: PLoS One. 2014 Sep 10;9(9):e107452. doi: 10.1371/journal.pone.0107452 (PMC4160247; doi:10.1371/journal.pone.0107452)
Supplement: Table S1 — Characteristics of pennate algal aggregates and sea ice stations investigated. (DOCX) [file pone.0107452.s002.docx]

**Table S1. Characteristics of pennate algal aggregates and sea ice stations investigated.**

| **Pennate Aggregates** | **P1** | **P2** | **P3** | **P4** | **P5** | **P6** |
| --- | --- | --- | --- | --- | --- | --- |
| **Station** | PS78/3_203 | PS78/3_209 | PS78/3_212 | PS80/3_224 | PS80/3_237 | PS80/3_323 |
| **Date (DD/MM/YYYY)** | 14.08.2011 | 17.08.2011 | 19.08.2011 | 09.08.2012 | 14.08.2012 | 04.09.2012 |
| **Latitude** | 85° 58.56' N | 86° 59.24' N | 88° 1.11' N | 84° 3.03' N | 83° 59.19' N | 81° 55.53' N |
| **Longitude** | 59° 25.16' E | 58° 29.37' E | 59° 58.53' E | 31° 6.83' E | 78° 6.20' E | 131° 7.72' E |
| **Size (cm)** | 1 | 5 | 10±5 | 10±5 | 7±5 | 2±1 |
| **Color** | White-yellowish | White-yellowish | White-yellowish | Green-brownish | Green-brownish | White-yellowish |
| **Environment** | Melt Pond | Melt Pond | Melt Pond | Below ice | Below ice | Melt Pond |
| **Sea Ice type** | FYI | FYI | FYI | FYI | FYI | FYI |
| **Melt Pond depth (m)** | 1 | n.d. | n.d.¥ | - | - | 0.35 |
| **Floating** | No | No | No | Yes | Yes | No |
| **Melt Pond coverage** | 50% | 50% | 50% | 40% | 20% | 10% |
| **Salinity (aggregate)** | 0-11 (11) | 9.6 | 1.9-5 (4.2) | 29.7 | 0.1-32(32) | 3.7-30.1 (28) |
| **Temperature (°C)** | -0.5 | n.d. | n.d. | 0.5 | n.d. | -1 |
| **Irradiance (µmol photons m^-2^ s^-1^)** | 160 | 211 | 130 | 99 | 52 | 78 |
| **Diatom species** | *Nitzschia, Pseudonitzschia, Navicula, Fragilariopsis, Chaetoceros, Coccolithophores* | *Chaetoceros, Fragilariopsis, Thalassiosira, Nitzschia,* | *Chaetoceros, Thalassiosira/Coscinodiscus, Nitzschia, Navicula, Fragilariopsis* | *Nitzschia, Navicula, Entomoneis, Fragilariopsis, Thalassiosira, Pleurosigma, Cylindrotheca* | *Nitzschia, Pseudonitzschia, Fossula arctica, Melosira arctica, Navicula, Entomoneis* | *Fragilariopsis, Navicula, Nitzschia* |
| **Grazing observed** | Ciliates | Ciliates | No | Amphipods and copepods | No | Ciliates |
| **POC (mg C L^-1 slurry^)** | 86 | 9 | 66 | 112 | 24 | 27 |
| **PON (µmol N mg POC^-1^)** | 8 | 5 | 5 | 10 | 8 | 2 |
| **C:N molar ratio** | 11 | 17 | 17 | 8 | 11 | 35 |
| **Chl *a* (µg Chl*a* mg POC^-1^)** | 2 | 0.6 | 0.3 | 9 | 5 | 0 |
| **C:Chl *a* ratio** | 505 | 1609 | 3306 | 110 | 212 | 66755 |
| **Chl*a*/CPE (%)** | 62 | 95 | 89 | 92 | 33 | 26 |
| **DOC (µmol C mg POC^-1^)** | n.d. | 7.4 | n.d. | 4.2 | 5.8 | 11.2 |
| **TEP (µg C mg POC^-1^)** | 8±8 | 171±115 | 10±16 | 1.5±0.3 | 1.5±1 | 4±1 |
| **TEP:POC** | 0.008±0.008 | 0.17±0.11 | 0.01±0.01 | 0.001±0.0003 | 0.001±0.001 | 0.004±0.001 |
| **NPP at 50µE (µg C mg POC^-1^ d^-1^)** | n.d. | n.d. | n.d. | 9.6 | 1.2 | 1.3 |
| **Bacterial counts (cells 10^9^ mg POC^-1^)** | 0.6 | 0.06 | 0.1 | 1.5 | 0.4 | 0.1 |
| **Bacterial POC (%)** | 1.8 | 0.2 | 0.3 | 4.4 | 1.2 | 0.2 |
| **Nitrate (µmol L^-1^)** | n.d. | n.d. | n.d. | 2.9 | 2.6 | 3.1 |
| **Phosphate (µmol L^-1^)** | n.d. | n.d. | n.d. | 0.3 | 0.2 | 0.01 |
| **Silicate (µmol L^-1^)** | n.d. | n.d. | n.d. | 1.2 | 1.3 | 0.0 |

All variables except nutrients were measured from an homogeneous algal slurry and normalized by POC. Nutrients were measured in the water surrounding the aggregates and are presented per volume of water.

Individual aggregate samples (a total of 11) for each aggregate type are labeled with P for pennate diatom aggregate and M for *Melosira* aggregates.

¥ Partially open melt pond.
