# Supplementary material for: Composition, Buoyancy Regulation and Fate of Ice Algal Aggregates in the Central Arctic Ocean
Source: PLoS One. 2014 Sep 10;9(9):e107452. doi: 10.1371/journal.pone.0107452 (PMC4160247; doi:10.1371/journal.pone.0107452)
Supplement: Table S2 — Characteristics of Melosira algal aggregates and sea ice stations investigated. (DOCX) [file pone.0107452.s003.docx]

**Table S2. Characteristics of *Melosira* algal aggregates and sea ice stations investigated.**

| **Melosira Aggregates** | **M1** | **M2** | **M3** | **M4** | **M5** |
| --- | --- | --- | --- | --- | --- |
| **Station** | PS80/3_224 | PS80/3_224 | PS80/3_224 | PS80/3_255 | PS80/3_349 |
| **Date (DD/MM/YYYY)** | 09.08.2012 | 09.08.2012 | 09.08.2012 | 20.08.2012 | 18.09.2012 |
| **Latitude** | 84° 3.03' N | 84° 3.03' N | 84° 3.03' N | 82° 40.24' N | 87° 56.01' N |
| **Longitude** | 31° 6.83' E | 31° 6.83' E | 31° 6.83' E | 109° 35.37' E | 61° 13.04' E |
| **Size (cm)** | 5 (filamentous) | 5±3 | 2±1 | 10±5 | 15±10 |
| **Color** | White-yellowish | White-yellowish | Green-brownish | White-yellowish | Green-brownish |
| **Environment** | Melt Pond | Melt Pond | Melt Pond | Melt Pond | Frozen in ice |
| **Sea Ice type** | FYI | FYI | FYI | FYI | MYI |
| **Melt Pond depth (m)** | 0.3¥ | 0.63 | 0.5 ¥ | 0.3 | Open |
| **Floating** | Yes | No | No | No | Yes |
| **Melt Pond coverage** | 40% | 40% | 40% | 40% | 20% |
| **Salinity (aggregate)** | 1.5-20.3 (6.9) | 0-32 (31.9) | 0.1-31(31) | 0.2-0.5 (0.5) | 31 |
| **Temperature (°C)** | 0.1 | -1.5 | 0.1 | 0.2 | 0.1 |
| **Irradiance (µmol photons m^-2^ s^-1^)** | 260 | 252 | 255 | 101 | 24 |
| **Diatom species** | *Melosira arctica, Nitzschia, Dinocysts* | *Melosira arctica, Nitzschia, Dinocysts* | *Melosira arctica, Nitzschia, Navicula, Fragilariopsis* | *Melosira arctica, Navicula, Nitzschia* | *Melosira arctica, Fragilariopsis, Cylindrotheca* |
| **Grazing observed** | Ciliates | Ciliates | No | Ciliates | Ciliates |
| **POC (mg C L^-1 slurry^)** | 7 | 49 | 132 | 16 | 11 |
| **PON (µmol N mg POC^-1^)** | 9 | 9 | 9 | 2 | 3 |
| **C:N molar ratio** | 10 | 10 | 9 | 40 | 28 |
| **Chl *a* (µg Chl*a* mg POC^-1^)** | 1.2 | 0.2 | 3 | 0.4 | 5 |
| **C:Chl *a* ratio** | 848 | 4623 | 337 | 2198 | 207 |
| **Chla/CPE (%)** | 82 | 96 | 98 | 94 | 77 |
| **DOC (µmol C mg POC^-1^)** | 23.3 | 7.2 | 4.6 | 35.4 | 17.3 |
| **TEP (µg C mg POC^-1^)** | 17±16 | 4±0.6 | 2±0.02 | 16±4 | 8±3 |
| **TEP:POC** | 0.02±0.01 | 0.004±0.001 | 0.002±0.0001 | 0.015±0.004 | 0.008±0.002 |
| **NPP at 50µE (µmg C mg POC^-1^ d^-1^)** | 0.013.3 | 0.014.1 | 0.001.5 | 0.002.2 | 0.154.9 |
| **Bacterial counts (cells 10^9^ mg POC^-1^)** | 0.6 | 0.1 | 0.5 | 0.1 | n.d. |
| **Bacterial POC (%)** | 1.8 | 0.4 | 1.7 | 0.2 | n.d. |
| **Nitrate (µmol L^-1^)** | 0.4 | 1.3 | 2.2 | 0.3 | 0.9 |
| **Phosphate (µmol L^-1^)** | 0.005 | 0.2 | 0.4 | 6.0 | 0.3 |
| **Silicate (µmol L^-1^)** | 1.6 | 2.6 | 20.7 | 0.7 | 1.4 |
|  |  |  |  |  |  |

All variables except nutrients were measured from an homogeneous algal slurry and normalized by POC. Nutrients were measured in the water surrounding the aggregates and are presented per volume of water.

Individual aggregate samples (a total of 11) for each aggregate type are labeled with P for pennate diatom aggregate and M for *Melosira* aggregates.

¥ Partially open melt pond.
